# Implementation science capacity building for immunization stakeholders in Africa: benefits and way forward

**DOI:** 10.11604/pamj.2025.50.38.44117

**Published:** 2025-01-30

**Authors:** Abdu Abdullahi Adamu, Duduzile Ndwandwe, Rabiu Ibrahim Jalo, Sidy Ndiaye, Jamal Ahmed, Charles Shey Wiysonge

**Affiliations:** 1 Polio Eradication Programme, World Health Organization Region Office for Africa, Djoue, BP 06, Brazzaville, Congo,; 2 Vaccine-Preventable Diseases Programme, World Health Organization Regional Office for Africa, Djoue, BP 06, Brazzaville, Congo,; 3 Cochrane South Africa, South African Medical Research Council, Francie van Zijl Drive, Parrow Valley, 7500, Cape Town, South Africa,; 4 Department of Community Medicine, Bayero University/Aminu Kano Teaching Hospital, along Zaria Road, Kano, Nigeria

**Keywords:** Routine immunization, implementation science, Immunization Agenda 2030, Africa, vaccinology, capacity building, education

## Abstract

The success of immunization programmes in maximizing the public health and economic benefits of vaccines hinges on the ability of stakeholders within countries at both national and subnational levels to implement effectively with equity as the Northern Star. The field of implementation science which emerged in response to know-do gaps, has several frameworks, models, and theories that can be used by immunization stakeholders to enhance vaccination efforts across diverse contexts. However, there is a need to up-skill immunization stakeholders in Africa with implementation science capacity. Existing immunization-related training on the continent are a low-hanging opportunity that can be leveraged to enhance core competencies like implementation theories and frameworks, implementation strategies, systems thinking, quality improvement, and process evaluation among stakeholders. We posit that strengthening the capacity and capability of immunization stakeholders in implementation science can lead to an improvement in its continuous usage within programme settings to solve contextual bottlenecks. Two pathways for achieving this are suggested in this article.

## Opinion

Vaccines have far-reaching multidimensional benefits [1]. However, immunization programmes in Africa are often faced with multiple implementation challenges that limit the public health benefits and economic impact of vaccines for individuals and societies [2]. It is now less than six years to the end of the Immunization Agenda 2030 (IA2030), yet, the World Health Organization (WHO) African Region is still lagging on key performance indicators [3].

Several delays contribute to this lag in immunization system performance in the African region. First is the delay in translating regional or continental immunization policies into national strategies. For example, some countries in the region are yet to include second-dose measles, inactivated polio, and human papillomavirus (HPV) vaccinations among other recommended vaccines in their national routine immunization schedules [4]. Second is the delay in translating national immunization strategies to practice guidelines that strengthen routine delivery of vaccination services. The effect of this is apparent in the suboptimal coverage of existing vaccines with the high number of zero-dose children across communities in the region [5]. Multiple factors can contribute to this, including the unavailability of vaccination services, or where available, missed opportunities for vaccination are high because services are not regular, not integrated, or both. In addition, catch-up activities often exclude older children who are unvaccinated or under-vaccinated thereby maintaining the immunity gaps. Another delay is related to the adoption of routine immunization at the service delivery level. This can be caused by a mixture of demand and supply side issues such as inadequate or untrained human resources for health, vaccine hesitancy, and others. Each of these delays reinforces a policy-to-practice chasm that is responsible for what is seemingly a vaccine availability paradox.

Conceptually, there are three subsystems of immunization stakeholders that are involved in translating policies into practice at the country level [6]. They include: National Immunization Technical Advisory Groups (NITAG) and public researchers in the immunization evidence synthesis subsystem; national and subnational Expanded Programme on Immunization (EPI) teams and partners in the immunization programme support subsystem; and various cadres of frontline health workers in public and private healthcare facilities as well as communities in the immunization service delivery subsystems [6]. These stakeholders can benefit from implementation science knowledge and skills to improve their functionality in tackling these delays to reduce immunization policy-to-practice gap.

The field of implementation science emerged specifically in response to know-do gaps in healthcare; whether these are research-to-policy or policy-to-practice gaps [7]. Implementation science focuses on the methods and strategies to promote systematic uptake of evidence-based interventions in routine practice settings [7]. Knowledge and skills in implementation science are important for immunization stakeholders because they can answer critical questions about 'what' contextual factors influence implementation outcomes of vaccination efforts in a particular setting [7]. Contexts are highly dynamic, especially at the subnational level and each setting is somewhat unique in terms of the complex interrelated factors (at play) that can affect vaccination efforts. Therefore, a good understanding of the role of contextual influence is needed to appropriately tailor vaccination approaches, and by this means, dissuade “one-size-fits-all” thinking and encourage programme differentiation and adaptation.

Importantly, implementation science is suitable for answering 'how' the implementation outcomes of vaccination efforts can be improved in real-life programme settings across diverse contexts [7]. Examples of such questions include: “how can vaccination services be scaled up through formal private health care providers and informal health care providers in a particular setting?”, and “how can vaccination services be integrated at every service delivery point to prevent missed opportunities among children that make an encounter with health facilities?” The Expert Recommendation for Implementing Change (ERIC) is a great tool for identifying implementation strategies that can be used to address implementation problems [8]. There are several implementation science frameworks, models, and theories that immunization stakeholders can use to guide how these questions are answered to ensure a holistic and analytic system-oriented approach [7]. This way, stakeholders can function collaboratively and cohesively as “vaccination strategists”, moving beyond individual roles as researchers, policymakers, service providers, or programme managers. In addition, implementation science can facilitate the de-implementation of ineffective or harmful strategies that affect vaccination efforts.

Existing immunization- or vaccinology-related capacity-building programmes in the African region are a low-hanging opportunity to expand implementation science knowledge and skills among immunization stakeholders. Core competencies like implementation theories and frameworks, implementation strategies, systems thinking, quality improvement, and process evaluation can be integrated into them.

One of such capacity-building programmes is NITAG training. The NITAG is an institutionalized body tasked with supporting immunization-related decision-making within countries, and as such, should be the first target for implementation of science skills development [9]. National Immunization Technical Advisory Groups have several objectives, part of which include guiding immunization research prioritization and suggesting evidence-based adjustments to programmes to improve implementation effectiveness. It is strategically important to train NITAG members on implementation science to support their countries with implementation research agenda setting and tracking. Other opportunities like WHO´s Mid-Level Management course on the Essential Programme on Immunization and the suites of specialized training developed by the Global Polio Eradication Initiative should be explored as well. It is worthwhile to integrate at least some level of implementation science competency in all immunization-related training at regional, national, and subnational levels.

Furthermore, various advanced vaccinology courses are conducted periodically to update stakeholders´ knowledge of vaccine development and emerging trends related to service delivery [10]. Some of the courses in the African region include the Vaccine for Africa Initiative (VACFA) Annual African Vaccinology Course, the University of the Witwatersrand (Wits) African Leadership in Vaccinology Expertise (ALIVE) Advance Vaccinology Course, and the South African Vaccination and Immunization Centre (SAVIC) Vaccinology Short Course in South Africa. In Uganda, there is the Centre for Vaccines and Immunization (EACVI) Vaccinology Course for Health Professionals. The Jenner Institute´s Vaccinology in Africa and International Vaccinology Course rotates in several countries. Whereas these courses cover biomedical science, clinical trials, and immunization programme structure among others, they rarely include implementation science competencies. This should be corrected immediately.

Additionally, there are several academic postgraduate training programmes on vaccinology in the African region [10]. Wits University, which is one of the institutions that offer a masters-level training in vaccinology also has a well-developed implementation science programme. Stronger collaboration between institutions that anchor these courses is encouraged to produce vaccinology experts for Africa who are well grounded in the science of implementation. Implementation science should be a core module for vaccinology students and implementation science students should have the option of choosing modules on vaccines and vaccinology as well.

**Figure 1:**
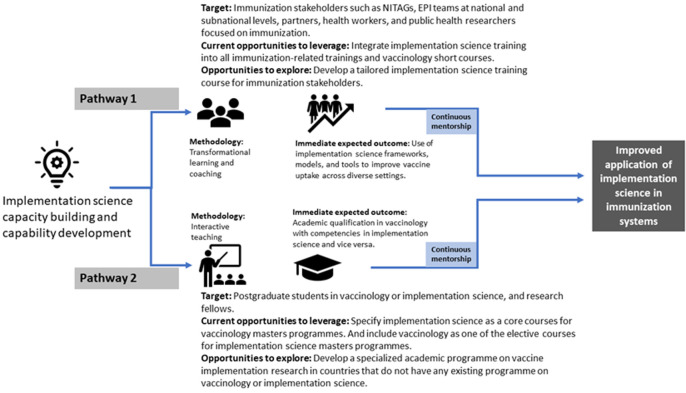
pathways for implementation science training among immunization stakeholders in Africa

It is time to transition to vaccinology 2.0 where the central focus is on implementation with equity. After all, vaccines are only useful for population health if they are administered to a significant proportion of people in the community. The World Health Organization and other partners can play an important role in motivating institutions to embark on this shift. Figure 1 is an illustration of the proposed pathways for advancing implementation science training among different types of immunization stakeholders. The hypothesis is that strengthening the capacity and capability of immunization stakeholders in implementation science can lead to an improvement in its continuous usage within programme settings to solve contextual bottlenecks. An important moderator to consider is continuous mentoring.

## Conclusion

The success of immunization programmes in Africa hinges on the ability of local stakeholders within countries at both national and subnational levels to implement effectively with equity as the Northern Star. So, as stakeholders untangle the multiple delays that weaken their immunization programmes, it is important to purposefully center policies and solutions around frequently marginalized populations in rural hard-to-reach communities, urban slums, or conflict-affected areas that are less likely to benefit from vaccination if the status quo is maintained. Unleashing the potential of implementation science in immunization systems can drive transformative improvement in system performance and, as such, it should be strongly encouraged.
